# Crystal structure and Hirshfeld surface analysis of (*E*)-1-(4-chloro­phen­yl)-2-[2,2-di­chloro-1-(4-fluoro­phen­yl)ethen­yl]diazene

**DOI:** 10.1107/S2056989019003657

**Published:** 2019-03-26

**Authors:** Namiq Q. Shikhaliyev, Sevim Türktekin Çelikesir, Mehmet Akkurt, Khanim N. Bagirova, Gulnar T. Suleymanova, Flavien A. A. Toze

**Affiliations:** a Organic Chemistry Department, Baku State University, Z. Xalilov str. 23, Az, 1148 Baku, Azerbaijan; b Department of Physics, Faculty of Sciences, Erciyes University, 38039 Kayseri, Turkey; c Department of Chemistry, Faculty of Sciences, University of Douala, PO Box 24157, Douala, Republic of Cameroon

**Keywords:** crystal structure, 4-chloro­phen­yl, 4-fluoro­phen­yl, face-to-face π-π stacking inter­action, Hirshfeld surface analysis

## Abstract

In the title compound, C_14_H_8_Cl_3_FN_2_, the planes of the 4-fluoro­phenyl ring and the 4-chloro­phenyl ring make a dihedral angle of 56.13 (13)°. In the crystal, mol­ecules are stacked in a column along the *a* axis *via* a weak C—H⋯Cl hydrogen bond and face-to-face π–π stacking inter­actions [centroid–centroid distances = 3.8615 (18) and 3.8619 (18) Å]. The crystal packing is further stabilized by short Cl⋯Cl contacts. The Hirshfeld surface analysis of the crystal structure indicates that the most important contributions for the crystal packing are from Cl⋯H/H⋯Cl (31.2%), H⋯H (14.8%), C⋯H/H⋯C (14.0%), F⋯H/H⋯F (12.8%), C⋯C (9.0%) and Cl⋯Cl (6.7%) inter­actions.

## Abstract 2

The dihedral angle between the 4-fluoro­phenyl ring and the 4-chloro­phenyl ring is 56.13 (13)°. In the crystal, mol­ecules are linked by C—H⋯Cl hydrogen bonds stacking in a column along the *a* axis. The crystal packing is further stabilized by face-to-face π–π stacking inter­actions between the centres of the similar aromatic rings of the adjacent mol­ecules.

## Chemical context   

Azo compounds provide ubiquitous motifs in synthetic chemistry and are widely used as organic dyes, indicators, mol­ecular switches, pigments, ligands, food additives, radical reaction initiators, therapeutic agents *etc.* (Gurbanov *et al.*, 2017 ▸; Maharramov *et al.*, 2018 ▸; Mahmudov *et al.*, 2019 ▸). Azo dyes are also convenient model compounds to study both *E*/*Z* isomerization and noncovalent inter­actions (Mahmudov *et al.*, 2015 ▸; Shixaliyev *et al.*, 2018 ▸). Thus, decorating the structure of dyes with tailored functionalities (noncovalent bond donor centres) can be a pivotal strategy for controlling and tuning their functional properties (Mahmudov *et al.*, 2017 ▸; Zubkov *et al.*, 2018 ▸). Herein we report the mol­ecular structure and noncovalent inter­actions in the title compound.
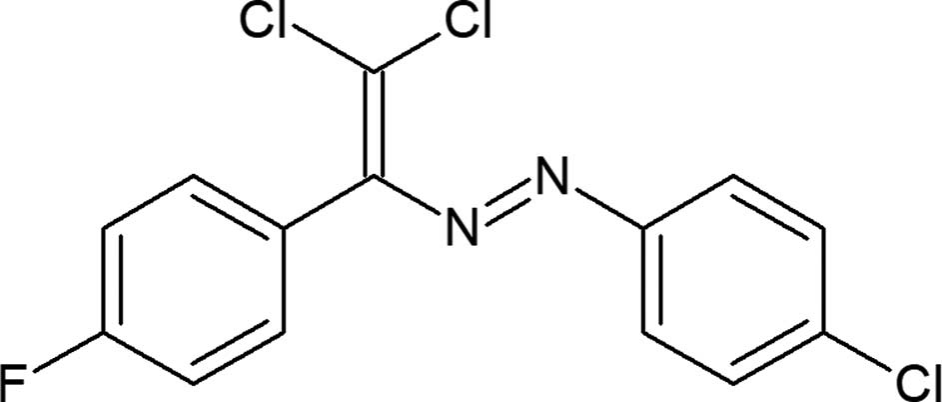


## Structural commentary   

**Figure 1:**
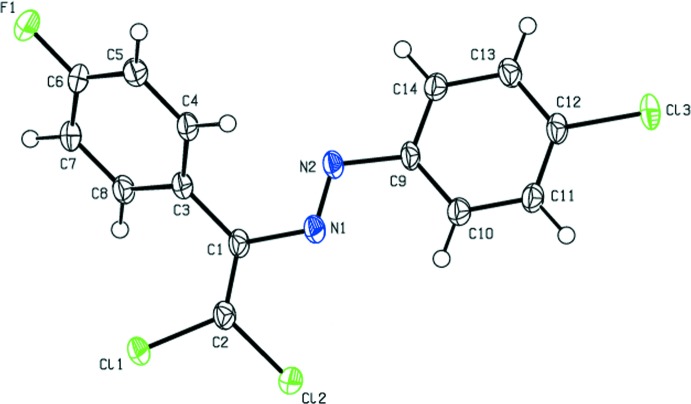
The mol­ecular structure of the title compound, with the atom-labelling scheme and 50% probability displacement ellipsoids.

The mol­ecular conformation of the title compound is not planar (Fig. 1 ▸); the planes of the 4-fluoro­phenyl ring and the 4-chloro­phenyl ring form a dihedral angle of 56.13 (13)°. The C4—C3—C1—N1, C8—C3—C1—C2, C3—C1—C2—Cl1, C3—C1—C2—Cl2, N1—C1—C2—Cl1, N1—C1—C2—Cl2, C1—N1—N2—C9 and N1—N2—C9—C14 torsion angles are 48.4 (4), 49.2 (4), −1.9 (4), 177.94 (19), 177.14 (18), −3.0 (3), 179.2 (2) and 175.9 (2)°, respectively.

## Supra­molecular features and Hirshfeld surface analysis   

**Figure 2:**
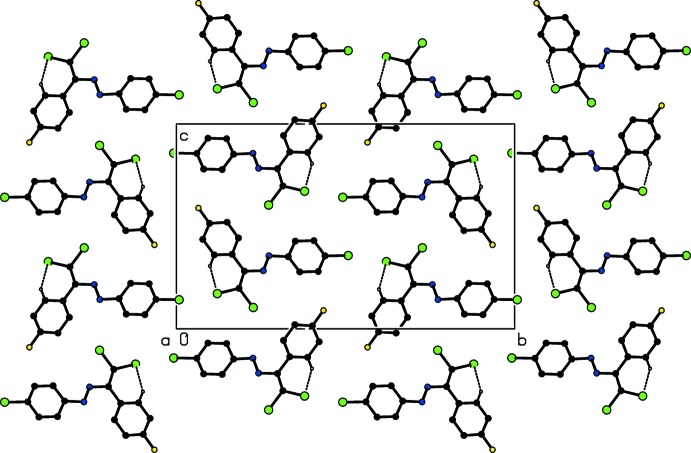
A packing diagram of the title compound, viewed along the *a* axis, showing the C—H⋯Cl inter­actions (dashed lines).

In the crystal, mol­ecules are linked by a weak C—H⋯Cl hydrogen bond (Table 1 ▸), forming a column along the *a* axis (Figs. 2 ▸ and 3 ▸). The column is further stabilized by face-to-face π–π stacking inter­actions; the centroid–centroid distances between the adjacent C3–C8 rings and between the adjacent C9–C14 rings are 3.8615 (18) and 3.8619 (18) Å, respectively. Moreover, the columns are linked by inter­molecular Cl⋯Cl short contacts, with distances of 3.3756 (11) and 3.3841 (11) Å (Table 2 ▸), forming a layer parallel to the *bc* plane (Fig. 2 ▸).

**Figure 4:**
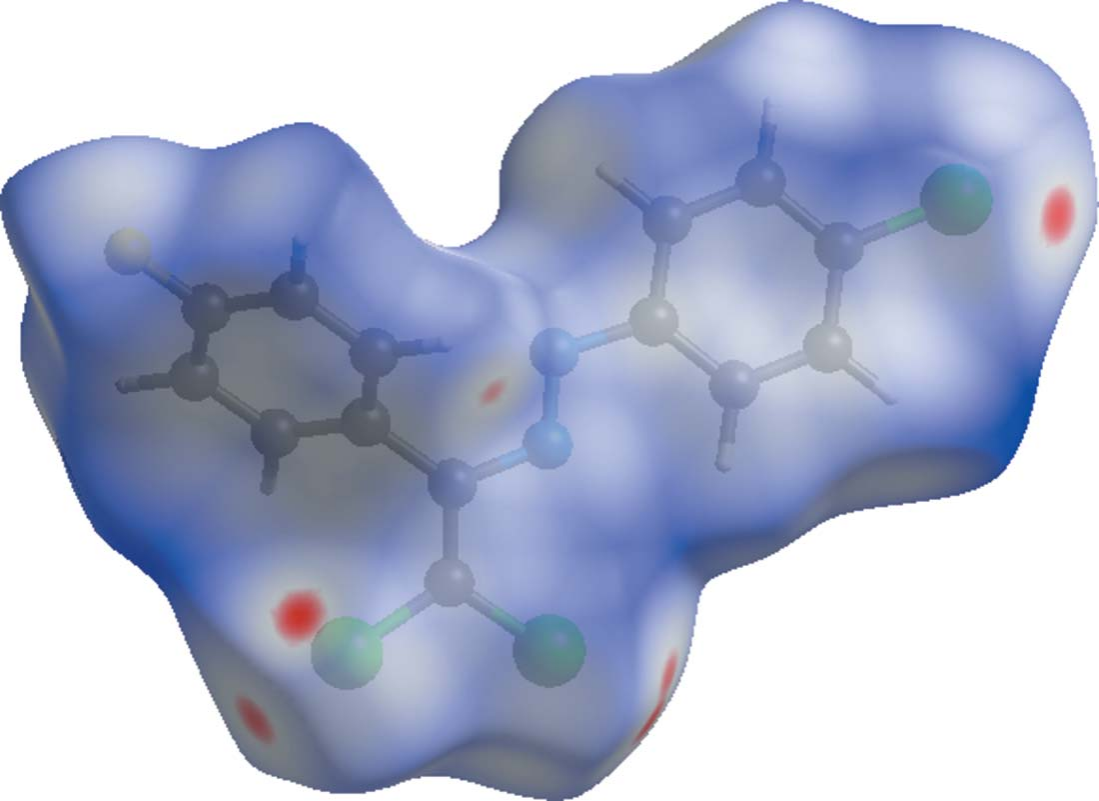
View of the Hirshfeld surface of the title compound plotted over *d*_norm_ in the range from −0.0941 to 1.4174 a.u.

**Figure 5:**
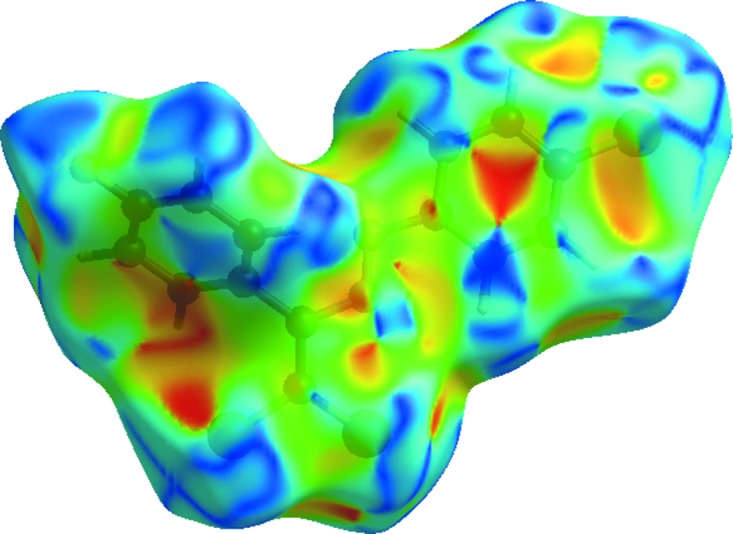
View of the Hirshfeld surface of the title compound plotted over shape index.

Hirshfeld surfaces and fingerprint plots were generated for the title compound using *CrystalExplorer* (McKinnon *et al.*, 2007 ▸). The Hirshfeld surface mapped over *d*_norm_ using a standard surface resolution with a fixed colour scale of −0.0941 (red) to 1.4174 a.u. (blue) is shown in Fig. 4 ▸. This plot was generated to qu­antify and visualize the inter­molecular inter­actions and to explain the observed crystal packing. The dark-red spots on the *d*_norm_ surface arise as a result of the C—H⋯Cl inter­action and short inter­atomic contacts (Tables 1 ▸ and 2 ▸), while the other weaker inter­molecular inter­actions appear as light-red spots. The shape index of the Hirshfeld surface is a tool to visualize the π–π stacking by the presence of adjacent red and blue triangles; if there are no adjacent red and/or blue triangles, then there are no π–π inter­actions. Fig. 5 ▸ clearly suggests that there are π–π inter­actions in the title compound.

**Figure 6:**
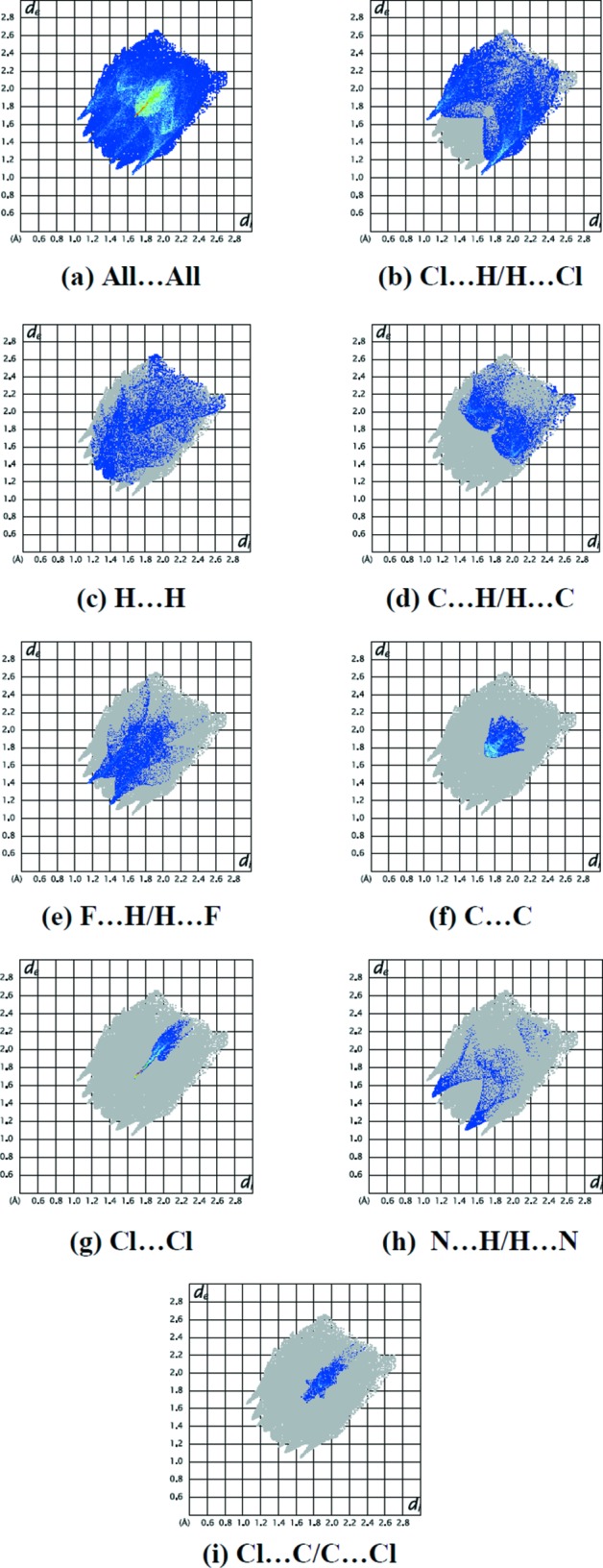
The full 2D fingerprint plots for the title compound, showing (*a*) all inter­actions, and those delineated into (*b*) Cl⋯H/H⋯Cl, (*c*) H⋯H, (*d*) C⋯H/H⋯C, (*e*) F⋯H/H⋯F, (*f*) C⋯C, (*g*) Cl⋯Cl, (*h*) N⋯H/H⋯N and (*i*) Cl⋯C/C⋯Cl inter­actions. The *d*_i_ and *d*_e_ values are the closest inter­nal and external distances (in Å) from given points on the Hirshfeld surface contacts.

**Figure 7:**
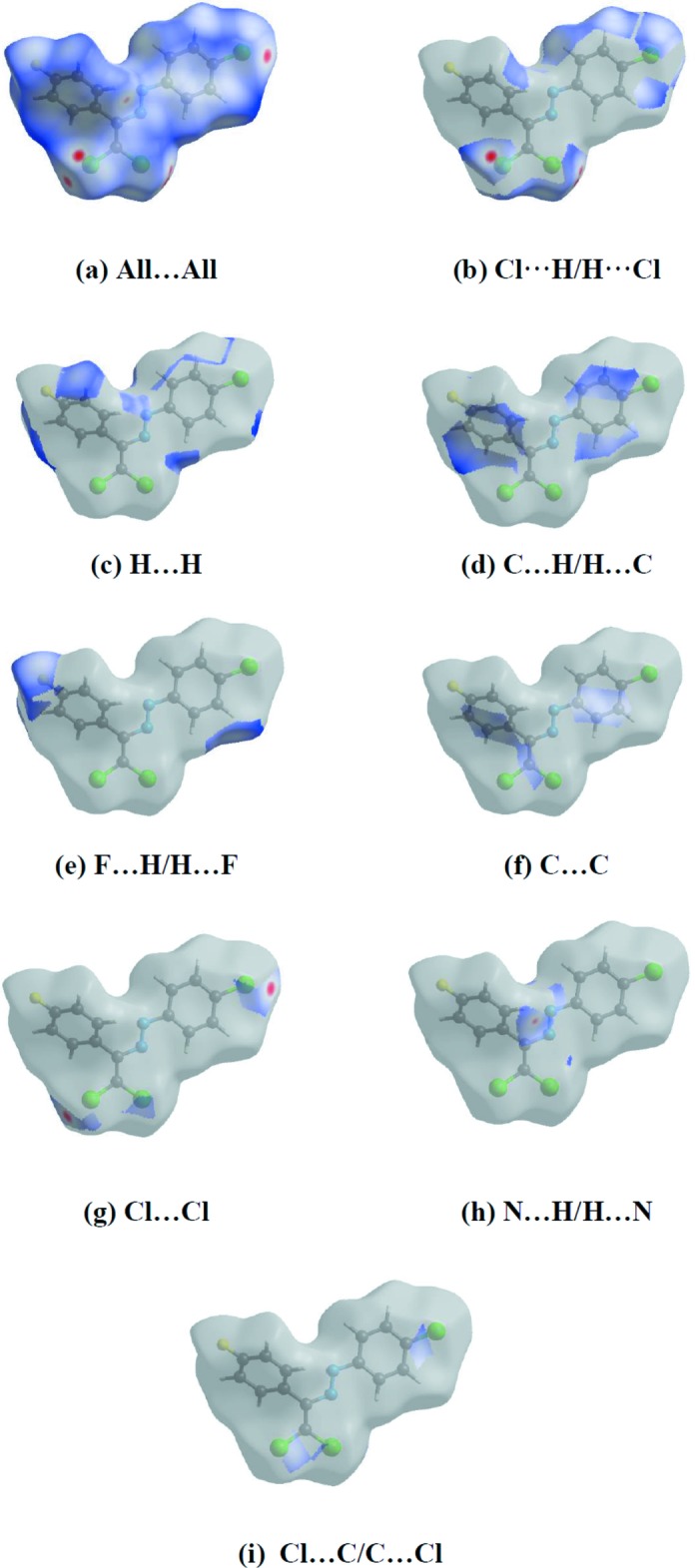
Hirshfeld surface representations with the function *d*_norm_ plotted onto the surface for (*a*) all inter­actions, (*b*) Cl⋯H/H⋯Cl, (*c*) H⋯H, (*d*) C⋯H/H⋯C, (*e*) F⋯H/H⋯F, (*f*) C⋯C, (*g*) Cl⋯Cl, (*h*) N⋯H/H⋯N and (*i*) Cl⋯C/C⋯Cl inter­actions.

The percentage contributions of the various contacts to the total Hirshfeld surface are shown in the 2D fingerprint plots in Fig. 6 ▸. The reciprocal Cl⋯H/H⋯Cl inter­actions appear as two symmetrical broad wings with *d*_e_ + *d*_i_ ≃ 2.7 Å and contribute 31.2% to the Hirshfeld surface (Fig. 6 ▸ *b*). The H⋯H inter­actions appear in the middle of the scattered points in the 2D fingerprint plots, with an overall contribution to the Hirshfeld surface of 14.8% (Fig. 6 ▸ *c*). The C⋯H/H⋯C inter­actions, with a 14.0% contribution, are present as bump symmetrical spikes at diagonal axes (Fig. 6 ▸ *d*). The F⋯H/H⋯F inter­actions, with a 12.8% contribution, are present as sharp symmetrical spikes at diagonal axes *d*_e_ + *d*_i_ ≃ 2.55 Å (Fig. 6 ▸ *e*). The C⋯C inter­actions appear in the middle of the scattered points in the 2D fingerprint plots with an overall contribution to the Hirshfeld surface of 9.0% (Fig. 6 ▸ *f*). The small percentage contributions from the other different inter­atomic contacts to the Hirshfeld surfaces are as follows: Cl⋯Cl (6.7%) (Fig. 6 ▸ *g*), N⋯H/H⋯N (3.4%) (Fig. 6 ▸ *h*), Cl⋯C/C⋯Cl (3.1%) (Fig. 6 ▸ *i*), N⋯C/C⋯N (2.8%), N⋯N (1.0%), Cl⋯N/N⋯Cl (0.8%), F⋯F (0.4%) and F⋯C/C⋯F (0.1%). Hirshfeld surface representations with the function *d*_norm_ plotted onto the surface for Cl⋯H/H⋯Cl, H⋯H, C⋯H/H⋯C, F⋯H/H⋯F, C⋯C, Cl⋯Cl, N⋯H/H⋯N and Cl⋯C/C⋯Cl inter­actions are shown in Fig. 7 ▸. The large number of Cl⋯H/H⋯Cl, H⋯H, C⋯H/H⋯C, F⋯H/H⋯F and C⋯C inter­actions suggest that van der Waals inter­actions and hydrogen bonding play the major roles in the crystal packing (Hathwar *et al.*, 2015 ▸).

## Database survey   

A search of the Cambridge Structural Database (CSD, Version 5.40, November 2018; Groom *et al.*, 2016 ▸) for structures having an (*E*)-1-(2,2-di­chloro-1-phenyl­vin­yl)-2-phenyldiazene unit gave 18 hits. Three compounds closely resemble the title compound, *viz*. 1-[2,2-di­chloro-1-(4-nitro­phen­yl)ethen­yl]-2-(4-fluoro­phen­yl)diazene (CSD refcode XIZREG; Atioğlu *et al.*, 2019 ▸), 1,1′-[methyl­enebis(4,1-phenyl­ene)]bis­[(2,2-di­chloro-1-(4-nitro­phen­yl)ethen­yl]diazene (LEQXIR; Shixaliyev *et al.*, 2018 ▸) and 1,1′-[methyl­enebis(4,1-phenyl­ene)]bis­{[2,2-di­chloro-1-(4-chloro­phen­yl)ethen­yl]dia­zene} (LEQXOX; Shixaliyev *et al.*, 2018 ▸). In XIZREG (Atioğlu *et al.*, 2019 ▸), mol­ecules are linked by a C—H⋯O hydrogen bond into a zigzag chain running along the *c* axis. The crystal packing is further stabilized by C—Cl⋯π, C—F⋯π and N—O⋯π inter­actions. In the crystal of LEQXIR, C—H⋯N and C—H⋯O hydrogen bonds and Cl⋯O contacts were found, and in LEQXOX, C—H⋯N and Cl⋯Cl contacts were observed.

## Synthesis and crystallization   

This dye was synthesized according to a reported method (Shixaliyev *et al.*, 2018 ▸). A 20 ml screw-necked vial was charged with dimethyl sulfoxide (10 ml), (*E*)-1-(4-chloro­phen­yl)-2-(4-fluoro­benzyl­idene)hydrazine (248 mg, 1 mmol), tetra­methyl­ethylenedi­amine (295 mg, 2.5 mmol), CuCl (2 mg, 0.02 mmol) and CCl_4_ (20 mmol, 10 equiv.). After 1–3 h (until thin-layer chromatography analysis showed complete consumption of the corresponding Schiff base), the reaction mixture was poured into a ∼0.01 *M* solution of HCl (100 ml, ∼pH = 2–3) and extracted with di­chloro­methane (3 × 20 ml). The combined organic phase was washed with water (3 × 50 ml), brine (30 ml), dried over anhydrous Na_2_SO_4_ and concentrated *in vacuo* with a rotary evaporator. The residue was purified by column chromatography on silica gel using appropriate mixtures of hexane and di­chloro­methane (3:1–1:1 *v*/*v*).

Red solid (yield 46%); m.p. 340–338 K. Analysis calculated (%) for C_14_H_8_Cl_3_FN_2_: C 51.02, H 2.45, N 8.50; found: C 49.95, H 2.43, N 8.47. ^1^H NMR (300 MHz, CDCl_3_): δ 7.15-7.17 (*m*, 4H), 7.42–7.45 (*d*, 2H, *J* = 9.21 Hz), 7.73–7.75 (*d*, 2H, *J* = 6.04 Hz). ^13^C NMR (75 MHz, CDCl_3_): δ 115.29, 115.58, 124.49, 127.46, 129.37, 130.43, 131.88, 131.99, 137.73, 151.13. ESI-MS: *m*/*z*: 330.44 [*M* + H]^+^.

## Refinement   

Crystal data, data collection and structure refinement details are summarized in Table 3 ▸. C-bound H atoms were constrained to an ideal geometry, with C—H = 0.95 Å and *U*_iso_(H) = 1.2*U*_eq_(C). Nine outliers (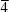,2,12; 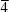,1,12; 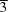,18,11; 2,21,1; 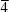,3,12; 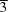,19,10; 0,13,17; 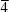,4,10; 2,20,0) were omitted in the final cycles of refinement.

## Supplementary Material

Crystal structure: contains datablock(s) I. DOI: 10.1107/S2056989019003657/is5510sup1.cif

Structure factors: contains datablock(s) I. DOI: 10.1107/S2056989019003657/is5510Isup2.hkl

CCDC reference: 1882554

Additional supporting information:  crystallographic information; 3D view; checkCIF report

## Figures and Tables

**Figure 3:**
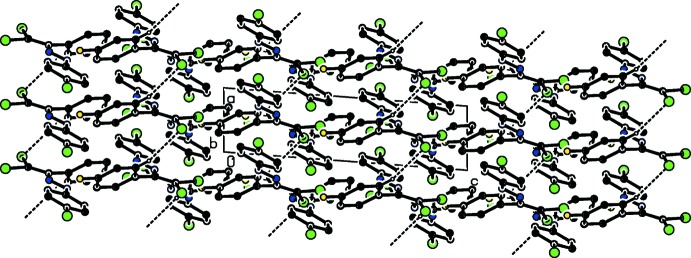
A packing diagram of the title compound, viewed along the *b* axis, showing the C—H⋯Cl inter­actions (dashed lines).

**Table 1:** Hydrogen-bond geometry (Å, °)

*D*—H⋯*A*	*D*—H	H⋯*A*	*D*⋯*A*	*D*—H⋯*A*
C8—H8⋯Cl1^i^	0.95	2.81	3.634 (3)	146

Symmetry code: (i) 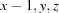.

**Table 2:** Summary of short inter­atomic contacts (Å) in the title compound

Contact	Distance	Symmetry operation
H4⋯N2	2.67	1 + *x*, *y*, *z*
Cl1⋯Cl3	3.3756 (11)	−*x*, − 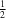 + *y*, 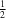 − *z*
Cl1⋯Cl3	3.3841 (11)	1 − *x*, − 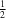 + *y*, 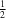 − *z*
Cl2⋯H14	3.03	1 + *x*, 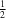 − *y*, − 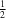 + *z*
H11⋯F1	2.81	*x*, 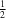 − *y*, − 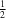 + *z*
H7⋯F1	2.67	1 − *x*, −*y*, 1 − *z*
F1⋯H11	2.84	1 + *x*, 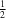 − *y*, 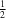 + *z*

**Table 3:** Experimental details

Crystal data	
Chemical formula	C_14_H_8_Cl_3_FN_2_
*M*_r_	329.57
Crystal system, space group	Monoclinic, *P*2_1_/*c*
Temperature (K)	100
*a*, *b*, *c* (Å)	3.8617 (8), 24.249 (5), 14.724 (3)
β (°)	94.30 (3)
*V* (Å^3^)	1374.9 (5)
*Z*	4
Radiation type	Synchrotron, λ = 0.80246 Å
μ (mm^−1^)	0.93
Crystal size (mm)	0.20 × 0.10 × 0.02

Data collection	
Diffractometer	Rayonix SX165 CCD
Absorption correction	Multi-scan (*SCALA*; Evans, 2006 ▸)
*T*_min_, *T*_max_	0.840, 0.970
No. of measured, independent and observed [*I* > 2σ(*I*)] reflections	20761, 2984, 2719
*R*_int_	0.115
(sin θ/λ)_max_ (Å^−1^)	0.640

Refinement	
*R*[*F* ^2^ > 2σ(*F* ^2^)], *wR*(*F* ^2^), *S*	0.053, 0.142, 1.05
No. of reflections	2984
No. of parameters	182
H-atom treatment	H-atom parameters constrained
Δρ_max_, Δρ_min_ (e Å^−3^)	0.59, −0.72

Computer programs: *Marccd* (Doyle, 2011 ▸), *iMosflm* (Battye *et al.*, 2011 ▸), *SHELXS97* (Sheldrick, 2008 ▸), *SHELXL2018* (Sheldrick, 2015 ▸), *ORTEP-3 for Windows* (Farrugia, 2012 ▸) and *PLATON* (Spek, 2009 ▸).

## supplementary crystallographic information

### Crystal data

**Table:**

C_14_H_8_Cl_3_FN_2_	*F*(000) = 664
*M**_r_* = 329.57	*D*_x_ = 1.592 Mg m^−^^3^
Monoclinic, *P*2_1_/*c*	Synchrotron radiation, λ = 0.80246 Å
*a* = 3.8617 (8) Å	Cell parameters from 600 reflections
*b* = 24.249 (5) Å	θ = 3.3–30.0°
*c* = 14.724 (3) Å	µ = 0.93 mm^−^^1^
β = 94.30 (3)°	*T* = 100 K
*V* = 1374.9 (5) Å^3^	Plate, orange
*Z* = 4	0.20 × 0.10 × 0.02 mm

### Data collection

**Table:**

Rayonix SX165 CCD diffractometer	2719 reflections with *I* > 2σ(*I*)
/f scan	*R*_int_ = 0.115
Absorption correction: multi-scan (*Scala*; Evans, 2006)	θ_max_ = 30.9°, θ_min_ = 3.3°
*T*_min_ = 0.840, *T*_max_ = 0.970	*h* = −4→4
20761 measured reflections	*k* = −30→31
2984 independent reflections	*l* = −18→18

### Refinement

**Table:**

Refinement on *F*^2^	Secondary atom site location: difference Fourier map
Least-squares matrix: full	Hydrogen site location: inferred from neighbouring sites
*R*[*F*^2^ > 2σ(*F*^2^)] = 0.053	H-atom parameters constrained
*wR*(*F*^2^) = 0.142	*w* = 1/[σ^2^(*F*_o_^2^) + (0.0557*P*)^2^ + 1.092*P*] where *P* = (*F*_o_^2^ + 2*F*_c_^2^)/3
*S* = 1.05	(Δ/σ)_max_ = 0.001
2984 reflections	Δρ_max_ = 0.59 e Å^−^^3^
182 parameters	Δρ_min_ = −0.72 e Å^−^^3^
0 restraints	Extinction correction: SHELXL2018 (Sheldrick, 2015), Fc^*^=kFc[1+0.001xFc^2^λ^3^/sin(2θ)]^-1/4^
Primary atom site location: difference Fourier map	Extinction coefficient: 0.026 (3)

### Special details

**Table:**

Geometry. All esds (except the esd in the dihedral angle between two l.s. planes) are estimated using the full covariance matrix. The cell esds are taken into account individually in the estimation of esds in distances, angles and torsion angles; correlations between esds in cell parameters are only used when they are defined by crystal symmetry. An approximate (isotropic) treatment of cell esds is used for estimating esds involving l.s. planes.

### Fractional atomic coordinates and isotropic or equivalent isotropic displacement parameters (Å^2^)

**Table:**

	*x*	*y*	*z*	*U*_iso_*/*U*_eq_	
Cl1	0.68912 (17)	0.12097 (2)	0.17209 (4)	0.0265 (2)	
Cl2	0.48467 (18)	0.22728 (2)	0.10431 (4)	0.0283 (2)	
Cl3	−0.18247 (18)	0.50802 (2)	0.35787 (5)	0.0318 (2)	
F1	0.5868 (5)	0.06509 (7)	0.58998 (10)	0.0386 (4)	
N1	0.3562 (6)	0.25699 (8)	0.28387 (14)	0.0246 (5)	
N2	0.2435 (6)	0.27366 (8)	0.35685 (14)	0.0230 (4)	
C1	0.4622 (7)	0.20110 (9)	0.28183 (16)	0.0225 (5)	
C2	0.5361 (7)	0.18506 (9)	0.19760 (16)	0.0237 (5)	
C3	0.4936 (7)	0.16469 (9)	0.36354 (16)	0.0232 (5)	
C4	0.6716 (7)	0.18329 (10)	0.44378 (16)	0.0249 (5)	
H4	0.7714	0.2191	0.4459	0.030*	
C5	0.7036 (7)	0.14978 (10)	0.52038 (16)	0.0283 (6)	
H5	0.8242	0.1622	0.5752	0.034*	
C6	0.5558 (8)	0.09804 (10)	0.51483 (17)	0.0287 (6)	
C7	0.3803 (7)	0.07791 (10)	0.43694 (17)	0.0280 (5)	
H7	0.2840	0.0418	0.4352	0.034*	
C8	0.3485 (7)	0.11196 (10)	0.36103 (17)	0.0243 (5)	
H8	0.2264	0.0992	0.3067	0.029*	
C9	0.1482 (7)	0.33066 (9)	0.35229 (16)	0.0225 (5)	
C10	0.1990 (7)	0.36475 (10)	0.27784 (16)	0.0251 (5)	
H10	0.3012	0.3504	0.2261	0.030*	
C11	0.1000 (7)	0.41943 (10)	0.27997 (16)	0.0257 (5)	
H11	0.1332	0.4430	0.2298	0.031*	
C12	−0.0490 (7)	0.43956 (10)	0.35640 (17)	0.0246 (5)	
C13	−0.0997 (7)	0.40658 (10)	0.43064 (17)	0.0255 (5)	
H13	−0.2002	0.4212	0.4824	0.031*	
C14	−0.0012 (7)	0.35157 (10)	0.42818 (16)	0.0248 (5)	
H14	−0.0358	0.3282	0.4784	0.030*	

### Atomic displacement parameters (Å^2^)

**Table:**

	*U*^11^	*U*^22^	*U*^33^	*U*^12^	*U*^13^	*U*^23^
Cl1	0.0363 (4)	0.0157 (3)	0.0273 (3)	0.0018 (2)	0.0006 (2)	−0.0038 (2)
Cl2	0.0408 (4)	0.0196 (3)	0.0243 (3)	0.0022 (2)	0.0017 (2)	0.0020 (2)
Cl3	0.0384 (4)	0.0125 (3)	0.0442 (4)	0.0020 (2)	0.0011 (3)	0.0002 (2)
F1	0.0622 (12)	0.0241 (8)	0.0290 (8)	0.0040 (8)	−0.0004 (7)	0.0087 (6)
N1	0.0333 (12)	0.0135 (9)	0.0266 (10)	−0.0008 (8)	0.0000 (8)	−0.0019 (7)
N2	0.0292 (11)	0.0128 (9)	0.0269 (10)	0.0004 (8)	0.0008 (8)	−0.0015 (7)
C1	0.0273 (13)	0.0123 (10)	0.0272 (11)	−0.0027 (9)	−0.0017 (9)	−0.0010 (8)
C2	0.0286 (13)	0.0146 (10)	0.0272 (11)	−0.0032 (9)	−0.0019 (9)	−0.0014 (8)
C3	0.0301 (13)	0.0143 (11)	0.0253 (11)	0.0017 (9)	0.0013 (9)	−0.0013 (8)
C4	0.0316 (14)	0.0149 (11)	0.0279 (11)	0.0016 (9)	0.0007 (10)	−0.0005 (9)
C5	0.0361 (15)	0.0214 (12)	0.0267 (11)	0.0039 (10)	−0.0023 (10)	−0.0023 (9)
C6	0.0407 (15)	0.0175 (11)	0.0280 (11)	0.0064 (10)	0.0037 (10)	0.0060 (9)
C7	0.0376 (15)	0.0143 (11)	0.0321 (12)	0.0007 (10)	0.0037 (10)	0.0016 (9)
C8	0.0291 (13)	0.0153 (11)	0.0285 (11)	0.0000 (9)	0.0013 (10)	−0.0017 (9)
C9	0.0288 (13)	0.0112 (10)	0.0269 (11)	0.0003 (9)	−0.0025 (9)	−0.0011 (8)
C10	0.0315 (14)	0.0176 (11)	0.0259 (11)	−0.0001 (9)	−0.0004 (9)	0.0001 (9)
C11	0.0332 (14)	0.0157 (11)	0.0277 (11)	−0.0010 (9)	−0.0020 (10)	0.0022 (9)
C12	0.0286 (13)	0.0132 (11)	0.0312 (12)	−0.0020 (9)	−0.0037 (10)	−0.0005 (9)
C13	0.0302 (13)	0.0165 (11)	0.0292 (11)	−0.0012 (9)	−0.0009 (9)	−0.0037 (9)
C14	0.0319 (14)	0.0175 (11)	0.0243 (11)	−0.0007 (9)	−0.0019 (9)	0.0013 (9)

### Geometric parameters (Å, º)

**Table:**

Cl1—C2	1.714 (2)	C6—C7	1.377 (4)
Cl2—C2	1.713 (2)	C7—C8	1.388 (3)
Cl3—C12	1.739 (2)	C7—H7	0.9500
F1—C6	1.363 (3)	C8—H8	0.9500
N1—N2	1.256 (3)	C9—C14	1.391 (3)
N1—C1	1.417 (3)	C9—C10	1.399 (3)
N2—C9	1.431 (3)	C10—C11	1.381 (3)
C1—C2	1.351 (3)	C10—H10	0.9500
C1—C3	1.490 (3)	C11—C12	1.390 (4)
C3—C8	1.395 (3)	C11—H11	0.9500
C3—C4	1.396 (3)	C12—C13	1.380 (3)
C4—C5	1.388 (3)	C13—C14	1.389 (3)
C4—H4	0.9500	C13—H13	0.9500
C5—C6	1.378 (4)	C14—H14	0.9500
C5—H5	0.9500		
			
N2—N1—C1	116.43 (19)	C8—C7—H7	121.0
N1—N2—C9	112.0 (2)	C7—C8—C3	120.9 (2)
C2—C1—N1	112.1 (2)	C7—C8—H8	119.6
C2—C1—C3	124.1 (2)	C3—C8—H8	119.6
N1—C1—C3	123.8 (2)	C14—C9—C10	120.4 (2)
C1—C2—Cl2	122.99 (19)	C14—C9—N2	115.8 (2)
C1—C2—Cl1	124.22 (18)	C10—C9—N2	123.9 (2)
Cl2—C2—Cl1	112.79 (14)	C11—C10—C9	119.6 (2)
C8—C3—C4	119.3 (2)	C11—C10—H10	120.2
C8—C3—C1	120.9 (2)	C9—C10—H10	120.2
C4—C3—C1	119.8 (2)	C10—C11—C12	119.2 (2)
C5—C4—C3	120.4 (2)	C10—C11—H11	120.4
C5—C4—H4	119.8	C12—C11—H11	120.4
C3—C4—H4	119.8	C13—C12—C11	122.0 (2)
C6—C5—C4	118.3 (2)	C13—C12—Cl3	118.9 (2)
C6—C5—H5	120.9	C11—C12—Cl3	119.10 (18)
C4—C5—H5	120.9	C12—C13—C14	118.7 (2)
F1—C6—C7	118.4 (2)	C12—C13—H13	120.6
F1—C6—C5	118.3 (2)	C14—C13—H13	120.6
C7—C6—C5	123.2 (2)	C13—C14—C9	120.1 (2)
C6—C7—C8	117.9 (2)	C13—C14—H14	119.9
C6—C7—H7	121.0	C9—C14—H14	119.9
			
C1—N1—N2—C9	179.2 (2)	C5—C6—C7—C8	−0.8 (4)
N2—N1—C1—C2	171.9 (2)	C6—C7—C8—C3	0.7 (4)
N2—N1—C1—C3	−9.0 (4)	C4—C3—C8—C7	−0.3 (4)
N1—C1—C2—Cl2	−3.0 (3)	C1—C3—C8—C7	179.5 (2)
C3—C1—C2—Cl2	177.94 (19)	N1—N2—C9—C14	175.9 (2)
N1—C1—C2—Cl1	177.14 (18)	N1—N2—C9—C10	−4.5 (4)
C3—C1—C2—Cl1	−1.9 (4)	C14—C9—C10—C11	0.0 (4)
C2—C1—C3—C8	−49.2 (4)	N2—C9—C10—C11	−179.6 (2)
N1—C1—C3—C8	131.8 (3)	C9—C10—C11—C12	0.0 (4)
C2—C1—C3—C4	130.5 (3)	C10—C11—C12—C13	0.3 (4)
N1—C1—C3—C4	−48.4 (4)	C10—C11—C12—Cl3	−178.57 (19)
C8—C3—C4—C5	−0.1 (4)	C11—C12—C13—C14	−0.6 (4)
C1—C3—C4—C5	−179.9 (2)	Cl3—C12—C13—C14	178.31 (19)
C3—C4—C5—C6	0.0 (4)	C12—C13—C14—C9	0.5 (4)
C4—C5—C6—F1	−180.0 (2)	C10—C9—C14—C13	−0.2 (4)
C4—C5—C6—C7	0.4 (4)	N2—C9—C14—C13	179.4 (2)
F1—C6—C7—C8	179.6 (2)		

### Hydrogen-bond geometry (Å, º)

**Table:**

*D*—H···*A*	*D*—H	H···*A*	*D*···*A*	*D*—H···*A*
C8—H8···Cl1^i^	0.95	2.81	3.634 (3)	146

Symmetry code: (i) *x*−1, *y*, *z*.
